# Prognostic factors of acute mesenteric ischemia in ICU patients

**DOI:** 10.1186/s12876-019-0999-8

**Published:** 2019-05-30

**Authors:** Martin Caluwaerts, Diego Castanares-Zapatero, Pierre-François Laterre, Philippe Hantson

**Affiliations:** Department of Intensive Care, Cliniques St-Luc, Université catholique de Louvain, Avenue Hippocrate, 10, 1200 Brussels, Belgium

**Keywords:** Arterial acute mesenteric ischemia, ICU patients, Mortality, Prognostic factors

## Abstract

**Background:**

The primary endpoint was to investigate the prognostic factors of acute mesenteric ischemia (AMI) in ICU patients.

**Methods:**

Retrospective observational, non-interventional, monocentric study of a cohort of 214 ICU patients with a confirmed diagnosis of arterial AMI.

**Results:**

We collected demographics, mortality, hospital stay, prior medical history, comorbidities, reasons for ICU admission, laboratory investigations, diagnostic procedures, therapy, severity scores. The 30-day mortality rate was 71% for the 214 patients with arterial AMI. The incidence of nonocclusive mesenteric ischemia was particularly high. AMI was a secondary diagnosis in 58% of patients. Half of the population was represented by surgical patients who mostly required an urgent procedure. The mortality rate was not different in the subgroup with aortic surgery. Three factors were associated with an increase or decrease in mortality: the maximal dose of vasopressors (VP) administered to the patient (OR = 1.20; 95%CI = 1.08–1.33; *p* <  0.001), arterial change in lactate values within the first 24 h of admission (OR = 1.24; 95%CI = 1.05–1.48; *p* = 0.012) and anticoagulation (OR = 0.19; 95%CI = 0.043–0.84; *p* = 0.029).

**Conclusions:**

Fatalities after AMI were related to a high incidence of multi-organ failure. The monitoring of arterial lactate appeared helpful to identify the patients with a poor prognosis.

## Background

Acute mesenteric ischemia (AMI) is often an underdiagnosed cause of acute abdomen and is still followed by a high mortality and morbidity rate, despite intensive care therapy [1–4]. AMI may be subdivided into four different types according to the pathophysiology. Embolic and thrombotic occlusion is responsible for approximately 2/3 of cases, while nonocclusive mesenteric ischemia (NOMI) and mesenteric venous thrombosis (MVT) represent each 1/6 of cases [5]. Early diagnosis is often impaired due to the lack of sensitivity and specificity of the clinical features and biomarkers. Computed tomography with angiography (CTA) is currently the “gold standard” diagnostic procedure in AMI, with an estimated sensitivity and specificity of 91 and 99%, respectively [6, 7]. However, CTA findings in the early stages of the affection may be more subtle, and the sensitivity is probably overestimated [1]. AMI may occur as a primary diagnosis but is also observed as a complication in ICU patients admitted with other comorbidities. To our best knowledge, few studies have looked specifically at the outcome of AMI in ICU patients [8].

The primary aim of our study was to assess the prognostic factors of arterial AMI in ICU patients admitted in a tertiary care center over a period of 17 years. Additional endpoint was to determine if AMI was the primary cause of admission in the ICU or if it was secondary to an underlying condition. Finally, we developed a score predicting AMI mortality in ICU patients.

## Methods

### Study design

This research was designed as a retrospective, observational, non-interventional, monocentric study, including ICU patients from a tertiary university hospital in Belgium. The inclusion period ranged from 2000 to 2017. Patients were included if they were admitted to the ICU and if they presented AMI during their ICU stay. Diagnosis of AMI was made using at least one of these four procedures: computed tomography angiography, surgery, arteriography or autopsy. The exclusion criteria were: age ≤ 18 years at diagnosis, invalidation of the diagnosis by surgery or autopsy, ischemia by extrinsic compression of mesenteric vessels (e.g., intestinal obstruction, volvulus, compressive tumor, abdominal compartment syndrome…), ≥ 50% of missing data in the patient’s record. Patients with acute mesenteric ischemia from venous origin were not considered.

### Data collection

The patients’ data were recovered from the ICU database with ICD9-CM codes corresponding to the pathology (codes: 557.0, 557.1 and 557.9). From a total of pre-selected 643 patients, 214 were ultimately included. Data collection included demographics, prior medical history (PMH), date of diagnosis, duration of hospital and ICU stay, 30-day and in-hospital mortality, recurrence of AMI after the initial episode, type of ischemia (occlusive or nonocclusive), reasons for admission in ICU (primary, secondary, medical versus surgical, urgent versus scheduled) and time from ICU admission to diagnosis.

Laboratory data were recorded at the time of diagnosis. For arterial lactate, we also looked at arterial lactate measurement 24 h after AMI diagnosis, total platelet count 24 h before AMI diagnosis and if platelets fell below the threshold of 100,000 per μl after AMI diagnosis.

For each patient, we reported the diagnostic procedures. If an autopsy was performed, we investigated if the diagnosis of AMI was revealed by this procedure. The CTA features were identified according to the radiologist’s protocol. We also reported if laparotomy contributed to the diagnosis, and time from diagnosis to laparotomy.

Concerning therapeutic issues, the following data were collected: maximal dose of vasopressors (norepinephrine) administered, whether the patient received vasopressors within the 24 h preceding diagnosis, or after diagnosis/surgery/arteriography, antimicrobial therapy, initiation of therapeutic anticoagulation after diagnosis, thrombolysis, embolectomy, stenting, laparoscopy, laparotomy.

At admission, Sequential Organ Failure Assessment (SOFA) [9] and Acute Physiology and Chronic Health Evaluation II (APACHE II) [10] scores were calculated.

The comorbidities developed during ICU stay were expressed as: new onset atrial fibrillation, acute pancreatitis, sepsis or septic shock (as defined in 2016 by Singer et al. [11]), heart failure (central venous oxygen saturation below 70% or need for inotropic support), mechanical ventilation 24 h prior to diagnosis, renal replacement therapy (RRT) after diagnosis. Regarding kidney failure, we also calculated RIFLE (Risk, Injury, Failure, Loss of kidney function and End-stage kidney disease) [12], AKIN (Acute Kidney Injury Network) [13] and KDIGO (Kidney Disease: Improving Global Outcomes) [14] scores, from the presence or absence of RRT, urine output at admission, the worst urine output during ICU stay, urine output at ICU discharge or death, and serum creatinine at AMI diagnosis.

### Statistical analysis

Statistical analyses were performed using SPSS 21 software (SPSS software [IBM Corp. 2011. IBM SPSS Statistics for Windows, Version 21.0. Armonk, NY, USA: IBM Corp]).

Categorical variables were reported as percentages and analyzed using Chi-squared test or Fisher’s exact test. Continuous variables were expressed as mean with standard deviation (SD) or median with interquartile range (IQR) according to the distribution. Unpaired t-test or Mann-Whitney U-test were used for comparisons.

The patients with arterial AMI were divided into two groups according to 30-day outcome. To assess the risk factors of 30-day mortality, a univariate logistic regression analysis was conducted. To build a multivariate logistic regression model, variables were selected using a method of forward elimination with p- value less than 0.20 for inclusion. The results were expressed as odds ratio (OR) with 95% confidence intervals [95%CI]. All tests were two-sided, with significance level of 5%.

Receiver operating characteristic (ROC) curves were generated to assess the accuracy of significant variables in predicting 30-day mortality in AMI patients.

### Ethics and consent

Institutional approval was provided by the Saint-Luc University Hospital Ethics Committee (Ref: 2015/11JUI/329) and our study complied with the Helsinki Declaration. To ensure confidentiality, patient data were anonymously recorded in the final database, in accordance with Belgian law. A waiver was obtained for written informed consent in view of the study’s retrospective design.

## Results

The detailed results are presented in Tables 1 and 2.

**Table 1:** Characteristics of patients with arterial AMI

Characteristics	Arterial AMI (*n* = 214)
Demographics	
Gender, male (%)	51
Age at diagnosis (years) (mean ± SD)	72 ± 13
Length of in-hospital stay (days) (median [IQR])	13 [4–30]
Length of ICU stay (days) (median [IQR])	4.5 [2–12]
In-hospital mortality rate (%)	71
30-day mortality rate (%)	68
Reason for ICU admission	
Main admission diagnosis (%)	42
Surgical reason (%)	50
Urgent reason (%)	90
PMH and comorbidities	
Arterial hypertension (%)	69
Hypercholesterolemia (%)	47
Diabetes (%)	20
Cardiomyopathy (%)	57
PMH of atrial fibrillation (%)	27
Peripheral artery disease (%)	44
PMH of cancer (%)	28
Chronic inflammatory disease (%)	18
Hypercoagulability (%)	6
PMH of surgery (%)	78
Laboratory findings	
CRP (mg/dl) (median [IQR])	18 [8–25]
Serum creatinine (mg/dl) (median [IQR])	2 [1.2–3]
LDH > 250 IU/l (%)	72
Leukocytes (× 10^3^/mm^3^) (median [IQR])	14.8 [8.8–22]
Neutrophils (×10^3^/mm^3^) (median [IQR])	12.6 [6.6–18]
Lymphocytes (× 10^3^/mm^3^) (median [IQR])	0.83 [0.5–1.18]
NLR (median [IQR])	14 [7.7–24]
INR (median [IQR])	1.4 [1.1–1.9]
Platelets 24 h before diagnosis (×10^3^/mm^3^) (median [IQR])	157 [88–277]
Platelets < 100,000/mm^3^ after diagnosis (%)	59
Arterial lactate at diagnosis (mmol/l) (median [IQR])	3.6 [2–7.5]
Arterial lactate 24 h after diagnosis (mmol/l) (median [IQR])	3.4 [1.7–8.7]
Diagnostic procedures	
Time from ICU admission to diagnosis (days) (median [IQR])	1 [0–4]
*CT-scan:*	
Visualization of vessel obstruction (%)	28
Hypo-enhancement of bowel wall (%)	64
Bowel wall thickening (%)	37
Dilated bowel segment (%)	21
Fat stranding (%)	2
Pneumatosis intestinalis (%)	37
Air in mesenteric vessels (%)	20
Arteriography performed (%)	8
*Laparotomy:*	
Not performed (%)	31
Performed < 24 h post-diagnosis (%)	66
Performed > 24 h post-diagnosis (%)	3
*Autopsy:*	
Autopsy performed (%)	17
Discovery of AMI during autopsy (%)	5
Therapeutic procedures	
Vasopressors in the 24 h preceding diagnosis (%)	51
Maximal dose of vasopressors (gamma/min) (median [IQR])	30 [10–53]
Vasopressors post-diagnosis (%)	81
Antimicrobial therapy (%)	90
Anticoagulation (%)	62
Thrombolysis or embolectomy (%)	5
Stenting (%)	5
Laparoscopy (%)	1
Laparotomy (%)	64
Scores	
SOFA (mean ± SD)	8.7 ± 5
APACHE II (mean ± SD)	25 ± 10
Comorbidities during ICU stay	
New onset of atrial fibrillation (%)	14
Acute pancreatitis (%)	8
Sepsis, septic shock (%)	58
S_CV_O_2_ < 70% or need for inotropic agent (%)	71
Mechanical ventilation 24 h before diagnosis (%)	47
RRT after diagnosis (%)	52
Urine output at ICU admission (ml/24 h) (mean ± SD)	928 ± 1011
Worst urine output during ICU stay (ml/24 h) (mean ± SD)	321 ± 523
Urine output at the end of ICU stay (ml/24 h) (mean ± SD)	638 ± 928
No AKI (%)	22
RIFLE R, AKIN and KDIGO 1 (%)	10
RIFLE I, AKIN and KDIGO 2 (%)	10
RIFLE F/L/E, AKIN and KDIGO 3 (%)	58

*AMI* acute mesenteric ischemia, *SD* standard deviation, *IQR* interquartile range, *ICU* intensive care unit, *PMH* prior medical history, *NLR* neutrophil-to-lymphocyte ratio, *RRT* renal replacement therapy, *AKI* acute kidney injury

**Table 2:** Comparison of arterial AMI according to 30-day outcome

Characteristics	Total (*n* = 214)	Survivors (*n* = 69)	Non survivors (*n* = 145)	*p- value*
Demographics				
Gender, male (%)	51	55	49	0.46
Age at diagnosis (years) (mean ± SD)	72 ± 13	72 ± 14	70 ± 13	0.16
Length of in-hospital stay (days) (median [IQR])	13 [5–30]	33 [16–78]	7 [3–19]	< 0.001
Reason for ICU admission				
Main admission diagnosis (%)	42	71	28	< 0.001
Surgical reason (%)	52	76	38	< 0.001
Urgent reason (%)	90	88	90	0.66
Aortic surgery (%)	17	25	14	0.050
PMH and comorbidities				
Arterial hypertension (%)	69	81	63	0.001
Hypercholesterolemia (%)	47	47	46	0.93
Diabetes (%)	20	15	23	0.16
Cardiomyopathy (%)	57	63	55	0.23
PMH of atrial fibrillation (%)	26	32	24	0.23
Peripheral artery disease (%)	44	49	41	0.32
PMH of cancer (%)	28	21	32	0.08
Chronic inflammatory disease (%)	18	18	17	0.91
Hypercoagulable state (%)	6	6	6	0.99
PMH of surgery (%)	78	74	80	0.3
Laboratory findings				
CRP (mg/dl) (median [IQR])	18 [8–25]	17 [6–24]	19 [8–28]	0.24
Serum creatinine (mg/dl) (median [IQR])	2 [1.2–3]	1.6 [1.1–2.8]	2.1 [1.3–3.1]	0.014
LDH > 250 IU/l (%)	72	58	78	0.02
Leukocytes (×10^3^/mm^3^) (median [IQR])	14.8 [8.8–22]	13 [7.4–20.3]	14.8 [9.5–21.5]	0.22
Neutrophils (× 10^3^/mm^3^) (median [IQR])	12.6 [6.6–18]	10.7 [6.2–18.1]	12.7 [6.9–18.2]	0.54
Lymphocytes (×10^3^/mm^3^) (median [IQR])	0.83 [0.5–1.18]	0.9 [0.55–1.14]	0.8 [0.54–1.24]	0.73
NLR (median [IQR])	14 [7.7–24]	10.4 [7.8–20.1]	14 [7.5–25.8]	0.36
INR (median [IQR])	1.4 [1.1–1.9]	1.2 [1.1–1.4]	1.4 [1.2–2]	< 0.001
Platelets 24 h before diagnosis (×10^3^/mm^3^) (median [IQR])	167 [88–277]	183 [103–263]	155 [79–269]	0.35
Platelets < 100,000/mm^3^ after diagnosis (%)	59	36	69	< 0.001
Arterial lactate at diagnosis (mmol/l) (median [IQR])	3.6 [2–7.5]	2.5 [1.4–3.8]	4.4 [2.6–9.4]	< 0.001
Arterial lactate 24 h after diagnosis (mmol/l) (median [IQR])	3.4 [1.7–8.7]	1.7 [1.3–2.3]	7.9 [3.3–15.4]	< 0.001
Time from ICU admission to diagnosis (days) (median [IQR])	1 [0–4]	0 [0–1]	1 [0–6]	< 0.001
*CT-scan:*				
Visualization of vessel obstruction (%)	28	36	24	0.1
Hypo-enhancement of bowel wall (%)	64	45	73	< 0.001
Bowel wall thickening (%)	37	45	33	0.11
Dilated bowel segment (%)	37	37	35	0.9
Fat stranding (%)	24	28	22	0.71
Pneumatosis intestinalis (%)	37	27	43	0.04
Air in mesenteric vessels (%)	20	15	22	0.25
Arteriography performed (%)	8	17	5	0.002
*Laparotomy:*				
Not performed (%)	31	22	35	–
Performed < 24 h post-diagnosis (%)	66	75	62	–
Performed > 24 h post-diagnosis (%)	3	3	3	–
*Autopsy:*				
Autopsy performed (%)	17	0	25	–
Discovery of AMI during autopsy (%)	5	0	5	–
Therapeutic procedures				
Vasopressors in the 24 h preceding diagnosis (%)	51	19	68	< 0.001
Maximal dose of vasopressors (gamma/min) (median [IQR])	30 [10–53]	7 [0–23]	38 [20–67]	< 0.001
Vasopressors post-diagnosis (%)	81	55	93	< 0.001
Antimicrobial therapy (%)	89	90	91	0.61
Anticoagulation (%)	62	81	53	< 0.001
Scores				
SOFA (mean ± SD)	8.7 ± 5	6 ± 4	10 ± 4.7	< 0.001
APACHE II (mean ± SD)	25 ± 10	19 ± 7.4	27 ± 10	< 0.001
Comorbidities during ICU stay				
New onset of atrial fibrillation (%)	14	12	15	0.48
Acute pancreatitis (%)	8	1	11	0.014
Sepsis, septic shock (%)	58	43	65	0.002
S_CV_O_2_ < 70% or need for inotropic agent (%)	71	47	83	< 0.001
Mechanical ventilation 24 h before diagnosis (%)	47	18	61	< 0.001
RRT after diagnosis (%)	52	31	60	< 0.001

*AMI* acute mesenteric ischemia, *SD* standard deviation, *IQR* interquartile range, *ICU* intensive care unit, *PMH* prior medical history, *NLR* neutrophil-to-lymphocyte ratio, *RRT* renal replacement therapy, *AKI* acute kidney injury

### Demographics and previous medical history

The 30-day mortality rate in the 214 included patients (51% men, mean age at diagnosis 72 years) was 71%, while in-hospital mortality was 68%. Looking at the mortality rate over consecutive periods (2000–2005, 2006–2011, 2012–2017), we were not able to document any difference.

Most of the patients had been admitted in the ICU for the acute management of a critical condition and 50% were surgical patients. The mortality rate was not different in the subgroup of patients with aortic surgery. Fatality was mainly observed in the group of septic patients, while survival was associated with elective abdominal surgery.

Concerning prior medical history and comorbidities, patients with arterial AMI presented frequently arterial hypertension, hypercholesterolemia and cardiomyopathy.

### Organs dysfunction at the time of diagnosis and during ICU stay

At the time of diagnosis, a high proportion of patients had already developed a significant degree of multiple organ dysfunction. This was related to the high incidence (58%) of sepsis or septic shock as the primary reason for ICU admission or during ICU stay. As a consequence, cardiac failure, according to a S_CV_O_2_ below 70% or a need for inotropic support, was present in 71% of the patients. On the whole, 51% of the patients had received vasopressors prior to diagnosis, and vasopressors requirement still increased up to 81% after diagnosis.

The incidence of acute kidney injury was particularly high, with 58% of the patients classified as RIFLE F/L/E, AKIN and KDIGO 3. Renal replacement therapy was required in 52% of the patients after diagnosis. 47% of the patients required mechanical ventilation 24 h before AMI diagnosis.

### Results of CTA investigations

Regarding the features of the CTA investigations, arterial occlusion was assessed in only 28% of the cases.

### Management of AMI

Due to the severity of multiple organ failure or to the pre-existing medical condition, a decision of withholding treatment was made for 35 patients and surgery was consequently not performed; all these patients died in the ICU. Resection of the proximal colon was needed in 77 patients due to the involvement of the ileocolic artery. Revascularization was only possible in 17 patients (11 patients by an endovascular procedure, and six patients during surgery). In this subgroup, intestinal resection was still required in five patients; one patient still developed progressive AMI despite revascularization and intestinal resection was not performed after laparotomy. The mortality in the subgroup with revascularization was 47%. In 24 patients, only an explorative laparotomy was performed (open and close procedure) and no patient survived. In 27 patients, a second laparotomy (redo-laparotomy) was performed; in 25 patients, a new intestinal resection was required, while intestinal ischemia did not progress in the two remaining cases without resection; eight patients survived at 30-day.

### Comparison survivors versus non survivors

Table 2 is comparing survivors and non survivors.

Survivors had a longer median in-hospital stay than non survivors (*p* <  0.001).

No PMH or comorbidity was significantly associated with outcome, except for arterial hypertension, which was associated with a better 30-day survival rate (*p* = 0.001).

Non survivors had a higher median serum creatinine concentration than survivors (*p* = 0.014).

**Fig. 1:**
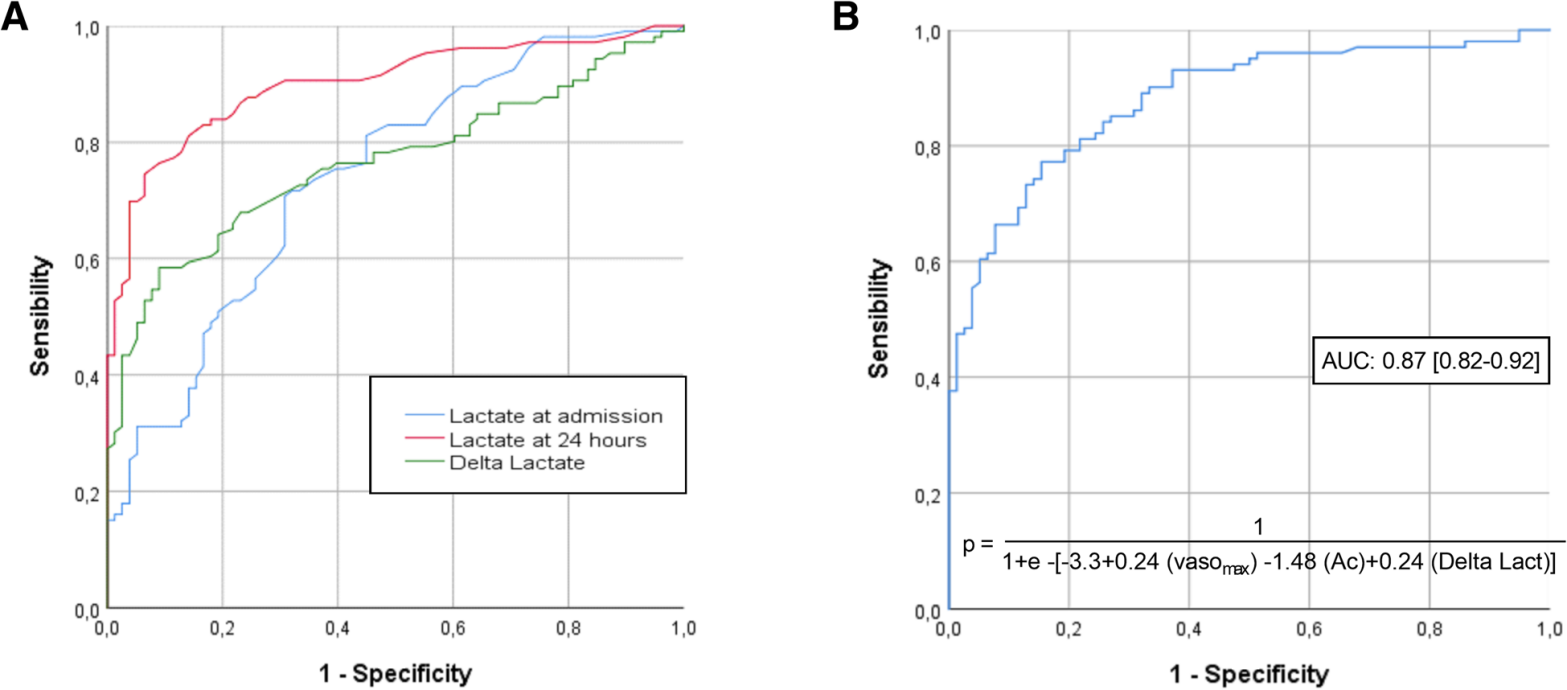
**a**. ROC curves assessing the performance of arterial lactate in predicting 30-day mortality in ICU patients with arterial AMI. AUC: area under the ROC curve.). Areas under the curves (AUC) are 0.722 (95%CI = 0.645–0.800), 0.892 (95%CI = 0.844–0.940) and 0.768 (95%CI = 0.698–0.837), respectively. **b.** ROC curve assessing the performance of the variable based on the multivariate logistic regression model: « 1.40 + 1.20 (Vaso max) + 0.19 (anticoagulation) + 1.24 (arterial delta lactate) », in predicting 30-day mortality in ICU patients with arterial AMI. AUC: area under the ROC curve. Vaso max: maximal dose of vasopressors

Survivors had a significantly lower arterial lactate concentration at diagnosis, but this difference was even more marked 24 h after diagnosis (p <  0.001). Figure 1a shows ROC curves assessing the prognostic performance of arterial lactate at diagnosis, 24 h after diagnosis, and difference between both (arterial delta lactate). The value of arterial lactate offering the best prognostic performance was 3.25. For arterial lactate 24 h after diagnosis, the optimal cutoff value was 3.65 mmol/l. For arterial delta lactate, the best Youden index was 0.85 mmol/l (increase of arterial lactate by 0.85 mmol/l during the first 24 h following diagnosis).

Concerning the relationship between CT features and 30-day outcome, hypo-enhancement of bowel wall and pneumatosis intestinalis were significantly associated with an increased mortality.

Use of vasoactive drugs and maximal dose of vasopressors were significantly higher among nonsurvivors, either in the 24 h preceding diagnosis or after diagnosis (*p* <  0.001). Anticoagulation was more often administered in survivors.

The patients who developed comorbidities (pancreatitis (*p* = 0.014), sepsis (*p* = 0.002), cardiac failure (p <  0.001)) or who required mechanical ventilation 24 h before diagnosis (p <  0.001) had a poorer prognosis.

### Univariate and multivariate analysis of prognostic factors

Independent variables were associated with 30-day outcome. To identify them, we performed a univariate analysis (left part of Table 3), followed by a multivariate logistic regression model (right part of Table 3) using a forward selection procedure, and based on arterial delta lactate.

**Table 3:** Risk factors of mortality from arterial AMI in ICU: univariate and multivariate analysis

Characteristics	OR	95%CI	*p-value*	Adj. OR	95%CI	*p-value*
Demographics						
Gender (male)	0.78	0.44–1.39	0.4	–	–	–
Age (years)	0.98	0.96–1.008	0.18	–	–	–
CT-scan						
Visualization of vessel obstruction	0.57	0.28–1.12	0.1	–	–	–
Hypo-enhancement of bowel wall	3.34	1.68–6.62	0.001	–	–	–
Bowel wall thickening	0.6	0.32–1.12	0.11	–	–	–
Dilated bowel segment	1	0.53–1.9	0.99	–	–	–
Fat stranding	0.87	0.43–1.78	0.71	–	–	–
Pneumatosis intestinalis	1.96	1.006–3.8	0.048	–	–	–
Air in mesenteric vessels	1.62	0.7–3.72	0.25	–	–	–
Pneumoperitoneum	0.73	0.35–1.54	0.41	–	–	–
PMH and comorbidities						
Arterial hypertension	0.42	0.21–0.83	0.014	–	–	–
Diabetes	1.74	0.8–3.80	0.16	–	–	–
Cardiomyopathy	0.7	0.38–1.26	0.23	–	–	–
PMH of atrial fibrillation	0.68	0.35–1.27	0.23	–	–	–
Peripheral artery disease	0.74	0.41–1.33	0.32	–	–	–
Laboratory findings						
CRP	1.01	0.99–1.035	0.24	–	–	–
Serum creatinine	1.25	1.001–1.55	0.049	–	–	–
LDH > 250 IU/l	2.56	1.38–4.86	0.003	–	–	–
Leukocytes	1.02	0.98–1.05	0.22	–	–	–
NLR	1.003	0.99–1.02	0.7	–	–	–
INR	7.1	2.8–17.6	< 0.001	–	–	–
Arterial lactate at diagnosis	1.27	1.14–1.41	< 0.001	–	–	–
Arterial lactate 24 h after diagnosis	2.1	1.56–2.75	< 0.001	–	–	–
Arterial delta lactate (after-before)	1.31	1.17–1.48	< 0.001	1.24	1.05–1.48	0.012
Platelets 24 h before diagnosis	1	0.99–1.002	0.99	–	–	–
Comorbidities and ICU management						
Time from ICU admission to diagnosis	1.1	1.02–1.18	0.011	–	–	–
New onset of atrial fibrillation during ICU stay	1.36	0.57–3.2	0.48	–	–	–
Maximal dose of vasopressors	1.26	1.17–1.35	< 0.001	1.20	1.08–1.33	< 0.001
S_CV_O_2_ < 70% or need for inotropic agent	5.42	2.6–11.26	< 0.001	–	–	–
Anticoagulation	0.26	0.13–0.51	< 0.001	0.19	0.043–0.84	0.029
Mechanical ventilation 24 h before diagnosis	7.45	3.65–15.2	< 0.001	–	–	–
SOFA score	1.26	1.12–1.4	< 0.001	–	–	–
APACHE II score	1.11	1.06–1.17	< 0.001	–	–	–

*OR* odds ratio, *95%CI*: 95% confidence interval, *Adj. OR* adjusted odds ratio, *PMH* prior medical history, *ICU* intensive care unit

This model assessed two factors associated with a poor 30-day outcome: maximal dose of vasoactive drug administered (OR = 1.20; 95%CI = 1.08–1.33; *p* <  0.001) and arterial delta lactate (OR = 1.24; 95%CI = 1.05–1.48; *p* = 0.012), and one factor associated with a better survival: anticoagulation (OR = 0.19; 95%CI = 0.043–0.84; *p* = 0.029). Using these three variables, we developed a 30-day risk of mortality score using dose of vasopressors, presence of anticoagulant therapy and arterial delta lactate (see Fig. 1a). A ROC curve of this new score was designed and is shown in Fig. 1b.

## Discussion

In the present study, the 30-day mortality rate for the 214 included patients was 71%, while in-hospital mortality was 68%. These mortality rates are congruent with those reported by the systematic review from Schoots et al. [7] and the meta-analysis from Adaba et al. [15], where in-hospital mortality rates were 71.6 and 63%, respectively.

Our mortality rate is also supported by a multicenter study by Leone et al. [8]. This study assessed risk factors of ICU mortality in AMI in France, and reported similar mortality rates, with 58% of ICU mortality and 63% of in-hospital mortality. With a 780-patient cohort, this study is currently the largest that assessed prognostic factors of AMI in ICU. However, it did not discriminate venous from arterial AMI. Our monocentric study extended on a longer period of time (17 years) than the study by Leone et al. (6 years). It assesses the mortality in an ICU belonging to a tertiary care center, with a proportion of patients referred from other ICUs in a critical condition.

Finally, the high mortality rate in our series is consistent with the particularly high incidence of NOMI. This form mainly occurs in patients presenting a severe and acute critical illness. The clinical presentation is often insidious and nonspecific, leading to delayed diagnosis [16, 17]. This explains also in our series the weak association of AMI with former or new onset atrial fibrillation.

Unlike in other studies [8, 18–20], age did not seem to be an independent risk factor of mortality in AMI.

We also underline the weak role of prior medical history or previously developed comorbidities on mortality. Only prior history of arterial hypertension seemed to improve the outcome. The reason for this speculative protective effect of arterial hypertension remains unclear, but could be related to a better preservation of pressure flow autoregulation in the splanchnic area [16].

The poor prognosis of AMI was also related to the number of organ dysfunctions as illustrated by the results of the univariate and multivariate analysis. It mainly refers to patients admitted with sepsis and who developed shock, acute kidney injury, and who required vasopressors and/or inotropes and mechanical ventilation. Regarding the use of vasoactive drugs, we cannot exclude its role in the increase of splanchnic vasoconstriction, even if vasopressin was not administered to our patients.

The prognostic value of arterial lactate concentration was also confirmed, and particularly the rise in arterial value within the first 24 h following diagnosis. Elevated serum lactate levels > 2 mmol/l was associated in irreversible intestinal ischemia in established diagnosis of AMI [21] While single lactate level is thought to be a strong predictor of subsequent organ dysfunction and mortality, it appears that lactate clearance is strongly associated with all-cause mortality in critically ill patients [22]. In the context of AMI, persistently elevated serum lactate is reflecting ongoing splanchnic hypoperfusion or progression of multiple organ failure. The diagnostic performance of lactate clearance may be even higher than in patients with sepsis. It has to be kept in mind that a normal value for arterial lactate does not exclude the diagnosis of AMI and that an isolated high lactate concentration may also reflect a late diagnosis.

On the other hand, anticoagulation initiated following diagnosis was associated with a better outcome in arterial AMI. While a continuous infusion of unfractionated heparin is a key treatment for venous mesenteric thrombosis, no benefit was clearly demonstrated to date in arterial AMI [23].

Derived from the multivariate analysis, we are proposing a predictive score of mortality that includes the maximal dose of vasopressors, the use of anticoagulation and the arterial delta lactate.

Regarding surgical approach, only a very few number of patients could benefit from revascularization. Intestinal resection was required for most of the cases and surgery, even if performed early, did not seem to improve prognosis. According to recent guidelines, more patients had second-look laparotomy over the last years of this survey. Damage control surgery (DCS) is now an important adjunct for critically-ill patients as it may allow to monitor the restoration of splanchnic perfusion [24].

Even if an autopsy was not performed in all deceased patients, the final cause of death appears to be in most of cases a multiple organ failure, often already present at the time of diagnosis. Eventually, in a significant number of medical files, a surgical abstention was probably decided due to the extent of the disease or to the patient’s condition following the development of multiple organ failure.

The limitations of this retrospective analysis have to be acknowledged.

First, this study does not permit to analyze the diagnostic accuracy of CTA, because according to our inclusion criteria, only patients with confirmed (and not only suspected) AMI were enrolled. The radiologist protocol did not include a reading grid, resuming every item contributing to AMI diagnosis. We deemed the CTA feature as absent if it was not explicitly mentioned in the radiologist protocol. In this study, the diagnostic and prognostic weight of bowel wall hypo-enhancement seems to be high. In ICU, the presence of an isolated pneumatosis intestinalis is not uncommon and can be related to other etiologies. We do not have data about false positive cases (e.g., pneumatosis intestinalis and AMI excluded by laparotomy). Finally, while significant progress has been made in CTA diagnosis over the last years, it appears unlikely that patients with NOMI could benefit from endovascular revascularization [25].

Second, the prevalence of some of the PMH or comorbidities may have been underestimated, because they have been reported from patients’ files. We estimate that most of PMH and comorbidities were reliably and consistently consigned in the medical files, making this bias minimal, except for hypercholesterolemia, chronic inflammatory disease and hypercoagulability. Concerning hypercholesterolemia, often not reported in the files, we attempted to limit the bias by searching for cholesterol-lowering agents in the medication history or for previous laboratory results showing hypercholesterolemia. The reason behind the underestimation of hypercoagulability and chronic inflammatory disease is that these conditions are often unknown in the setting of the ICU.

Third, the value arterial lactate 24 h after diagnosis is probably underestimated. Indeed, rapidly deceased patients (in less than 24 h) would probably have, considering our results, increased or persistently high concentrations of arterial lactate. The consideration of this bias strengthens the prognostic value of arterial lactate 24 h after diagnosis and arterial delta lactate, two variables identified as associated with poor outcome. Patients with AMI as main ICU admission diagnosis often do not have data about total platelet count 24 h prior to diagnosis. It could have led to an underestimation of the platelet count, because these patients probably had a mean platelet count higher than patients who were admitted in ICU for another reason, the latter in whom platelets could have decreased from other conditions.

Finally, as the period of data analysis extended over 17 years, even if the medical staff remained unchanged, significant changes may have occurred in diagnostic and therapeutic procedures over time.

## Conclusions

Fatalities after AMI were related to a high incidence of multi-organ failure. The monitoring of arterial lactate appeared helpful to identify the patients with a poor prognosis.

## Data Availability

To ensure that data confidentiality is not compromised, the dataset supporting the results of this article will not be integrated in the manuscript. The datasets are available from the corresponding author on reasonable request.

## Abbreviations

AMI: Acute mesenteric ischemia
CTA: Computed tomography with angiography
ICU: Intensive care unit
MVT: Mesenteric venous thrombosis
NOMI: Nonocclusive mesenteric ischemia
PMH: Prior medical history
RRT: Renal replacement therapy

## References

[1] Kärkkäinen JM, Acosta S (2017). Acute mesenteric ischemia (part I) - incidence, etiologies, and how to improve early diagnosis. Best Pract Res Clin Gastroenterol.

[2] Clair DG, Beach JM (2016). Mesenteric Ischemia. N Engl J Med.

[3] Kassahun WT, Schulz T, Richter O, Hauss J (2008). Unchanged high mortality rates from acute occlusive intestinal ischemia: six year review. Langenbeck's Arch Surg.

[4] Schoots IG, Koffeman GI, Legemate DA, Levi M, van Gulik TM (2004). Systematic review of survival after acute mesenteric ischaemia according to disease aetiology. Br J Surg.

[5] Acosta S (2010). Epidemiology of mesenteric vascular disease: clinical implications. Semin Vasc Surg.

[6] Cudnik MT, Darbha S, Jones J, Macedo J, Stockton SW, Hiestand BC (2013). The diagnosis of acute mesenteric ischemia: a systematic review and meta-analysis. Acad Emerg Med.

[7] Menke J (2010). Diagnostic accuracy of multidetector CT in acute mesenteric ischemia: systematic review and meta-analysis. Radiology.

[8] Leone M, Bechis C, Baumstarck K, Ouattara A, Collange O, Augustin P, Annane D, Arbelot C, Asehnoune K, Baldési O, Bourcier S, Delapierre L, Demory D, Hengy B, Ichai C, Kipnis E, Brasdefer E, Lasocki S, Legrand M, Mimoz O, Rimmelé T, Aliane J, Bertrand PM, Bruder N, Klasen F, Friou E, Lévy B, Martinez O, Peytel E, Piton A, Richter E, Kamel T, Vogler MC, Wallet F, Boufi M, Allaouchiche B, Constantin JM, Martin C, Jaber S, Lefrant JY (2015). Outcome of acute mesenteric ischemia in the intensive care unit: a retrospective, multicenter study of 780 cases. Intensive Care Med.

[9] Vincent JL, Moreno R, Takala J, Willatts S, De Mendonça A, Bruining H, Reinhart CK, Suter PM, Thijs LG (1996). The SOFA (Sepsis-related organ failure assessment) score to describe organ dysfunction/failure. On behalf of the working group on Sepsis-related problems of the European Society of Intensive Care Medicine. Intensive Care Med.

[10] Knaus WA, Draper EA, Wagner DP, Zimmerman JE (1985). APACHE II: a severity of disease classification system. Crit Care Med.

[11] Singer M, Deutschman CS, Seymour CW, Shankar-Hari M, Annane D, Bauer M, Bellomo R, Bernard GR, Chiche JD, Coopersmith CM, Hotchkiss RS, Levy MM, Marshall JC, Martin GS, Opal SM, Rubenfeld GD, van der Poll T, Vincent JL, Angus DC (2016). The third international consensus definitions for Sepsis and septic shock (Sepsis-3). JAMA.

[12] Bellomo R, Ronco C, Kellum JA, Mehta RL, Palevsky P (2004). Acute Dialysis quality initiative workgroup. Acute renal failure - definition, outcome measures, animal models, fluid therapy and information technology needs: the second international consensus conference of the acute Dialysis quality initiative (ADQI) group. Crit Care.

[13] Mehta RL, Kellum JA, Shah SV, Molitoris BA, Ronco C, Warnock DG (2007). Acute kidney injury network: report of an initiative to improve outcomes in acute kidney injury. Crit Care.

[14] Group AKIW (2012). KDIGO clinical practice guideline for acute kidney injury. Kidney Int Suppl.

[15] Adaba F, Askari A, Dastur J, Patel A, Gabe SM, Vaizey CJ (2015). Mortality after acute primary mesenteric infarction: a systematic review and meta-analysis of observational studies. Color Dis.

[16] Al-Diery H, Phillips A, Evennett N, Pandanaboyana S, Gilham M, Windsor JA. The pathogenesis of nonocclusive mesenteric ischemia: implications for research and clinical practice. J Intensive Care Med. 2018 Jan 1:885066618788827. 10.1177/0885066618788827. 10.1177/0885066618788827 30037271

[17] Yukaya T, Saeki H, Taketani K, Ando K, Ida S, Kimura Y, Oki E, Yasuda M, Morita M, Shirabe K, Maehara Y (2014). Clinical outcomes and prognostic factors after surgery for non occlusive mesenteric ischemia: a multicenter study. J Gastroenterol Surg.

[18] Acosta-Merida MA, Marchena-Gomez J, Hemmersbach-Miller M, Roque-Castellano C, Hernandez-Romero JM (2006). Identification of risk factors for perioperative mortality in acute mesenteric ischemia. World J Surg.

[19] Haga Y, Odo M, Homma M, Komiya K, Takeda K, Koike S, Takahashi T, Hiraka K, Yamashita H, Tanakaya K (2009). New prediction rule for mortality in acute mesenteric ischemia. Digestion.

[20] Aliosmanoglu I, Gul M, Kapan M, Arikanoglu Z, Taskesen F, Basol O, Aldemir M (2013). Risk factors effecting mortality in acute mesenteric ischemia and mortality rates: a single center experience. Int Surg.

[21] Nuzzo A, Maggiori L, Ronot M, Becq A, Plessier A, Gault N, Joly F, Castier Y, Vilgrain V, Paugam C, Panis Y, Bouhnik Y, Cazals-Hatem D, Corcos O (2017). Predictive factors of intestinal necrosis in acute mesenteric ischemia: prospective study from an intestinal stroke center. Am J Gastroenterol.

[22] Zhang Z, Xu X (2014). Lactate clearance is a useful biomarker for the prediction of all-cause mortality in critically ill patients: a systematic review and meta-analysis. Crit Care Med.

[23] Luther B, Mamopoulos A, Lehmann C, Klar E (2018). The ongoing challenge of acute mesenteric ischemia. Vasc Med.

[24] Bala M, Kashuk J, Moore EE, Kluger Y, Biffl W, Gomes CA, Ben-Ishay O, Rubinstein C, Balogh ZJ, Civil I, Coccolini F, Leppaniemi A, Peitzman A, Ansaloni L, Sugrue M, Sartelli M, Di Saverio S, Fraga GP, Catena F (2017). Acute mesenteric ischemia: guidelines of the world Society of Emergency Surgery. World J Emerg Surg.

[25] Cudnik MT, Darbha S, Jones J, Macedo J, Stockton SW, Hiestand BC (2013). The diagnosis of acute mesenteric ischemia:a systematic review andmeta-analysis. Acad Emerg Med.

